# *Leishmania mexicana* Infection Induces IgG to Parasite Surface Glycoinositol Phospholipids that Can Induce IL-10 in Mice and Humans

**DOI:** 10.1371/journal.pntd.0002224

**Published:** 2013-05-09

**Authors:** Laurence U. Buxbaum

**Affiliations:** 1 Philadelphia Research and Education Foundation, Philadelphia, Pennsylvania, United States of America; 2 VA Medical Center, Philadelphia, Pennsylvania, United States of America; 3 Department of Medicine, Division of Infectious Diseases, University of Pennsylvania School of Medicine, Philadelphia, Pennsylvania, United States of America; Hospital Universitário, Brazil

## Abstract

Infection with the intracellular protozoan parasite *Leishmania mexicana* causes chronic disease in C57BL/6 mice, in which cutaneous lesions persist for many months with high parasite burdens (10^7^–10^8^ parasites). This chronic disease process requires host IL-10 and FcγRIII. When *Leishmania* amastigotes are released from cells, surface-bound IgG can induce IL-10 and suppress IL-12 production from macrophages. These changes decrease IFN-γ from T cells and nitric oxide production in infected cells, which are both required for *Leishmania* control. However, antibodies targets and the kinetics of antibody production are unknown. Several groups have been unsuccessful in identifying amastigote surface proteins that bind IgG. We now show that glycoinositol phospholipids (GIPLs) of *L. mexicana* are recognized by mouse IgG1 by 6 weeks of infection, with a rapid increase between 12 and 16 weeks, consistent with the timing of chronic disease in C57BL/6 mice vs. healing in FcγRIII-deficient mice. A single prominent spot on TLC is recognized by IgG, and the glycolipid is a glycosyl phosphatidylinositol containing a branched mannose structure. We show that the lipid structure of the GIPL (the *sn*-2 fatty acid) is required for antibody recognition. This GIPL is abundant in *L. mexicana* amastigotes, rare in stationary-phase promastigotes, and absent in *L. major*, consistent with a role for antibodies to GIPLs in chronic disease. A mouse monoclonal anti-GIPL IgG recognizes GIPLs on the parasite surface, and induces IL-10 from macrophages. The current work also extends this mouse analysis to humans, finding that *L. mexicana*-infected humans with localized and diffuse cutaneous leishmaniasis have antibodies that recognize GIPLs, can bind to the surface of amastigotes, and can induce IL-10 from human monocytes. Further characterization of the target glycolipids will have important implications for drug and vaccine development and will elucidate the poorly understood role of glycolipids in the immunology of infections.

## Author Summary

*Leishmania mexicana* is a single-celled parasite that causes chronic skin disease in humans and mice. Antibodies on the surface of parasites lead to the production of a protein called interleukin-10 (IL-10), which blocks an effective immune response needed to kill parasites and resolve skin lesions. In mice, IL-10 is required to maintain chronic, non-healing lesions. Parasite surface targets of these antibodies have not been identified. Using biochemical and immunologic techniques, we have shown that antibodies bind to parasite surface glycolipids (molecules with sugars that are anchored to the membrane by lipids), rather than to protein targets. We have determined some basic structural features of these glycolipids and shown that antibodies to them bind the surface of parasites and can induce IL-10 from mouse cells. We have extended this work to humans by showing that people infected with this parasite also make antibodies that bind to these glycolipids and to the surface of parasites, and that can induce IL-10 from human white blood cells. Further characterization of these glycolipids may have important implications for the development of a drug or vaccine for this and related parasite infections, and may shed light on poorly understood immunologic pathways by which glycolipids induce antibody responses.

## Introduction

*Leishmania* is an intracellular protozoan parasite that causes 2 million new infections yearly and is a major cause of death worldwide [1]. Drug toxicity and the development of resistance have made leishmaniasis an ever-challenging set of diseases [2], [3], [4]. While a vaccine is likely the best way to deal with leishmaniasis, development has been hampered by our lack of understanding of factors needed to induce long-lasting cell-mediated immunity. Infections in which antibodies are protective, caused by bacteria such as *Streptococcus pneumoniae*, and many viral infections such as hepatitis B, have yielded successful vaccines [5], [6]. However, *Leishmania* are able to hide from antibodies in an intracellular location. When *Leishmania* amastigote stages, found in the mammalian host, are released from the cell to parasitize new host cells, the parasite is bound by antibodies and utilizes mechanisms to prevent lysis by complement [7], [8]. In fact, not only are antibodies not helpful, they can be pathogenic [9], [10], [11].

The immune response to the better-studied *L. major* infection is well explained by the Th1/Th2 paradigm, with IFN-γ-associated Th1 responses being protective and IL-4-associated Th2 responses leading to susceptibility. Non-healing infections such as those caused by *L. mexicana* complex parasites do not fit well into this explanation [12]. Mice that lack IL-4 (a key cytokine of Th2 responses) have chronic infection with *L. mexicana* and *L. amazonensis* [13], [14], and mice that lack IL-12 (the master cytokine driving Th1 responses) are just as susceptible as wild-type B6 mice to *L. mexicana* [15]. We therefore looked for other explanations and found that that IL-10 is required for chronic disease with *L. mexicana* infection [14]. C57BL/6 (B6) mice lacking IL-10 resolve infection with a protective IFN-γ response. IL-10 exerts multiple immunosuppressive functions such as decreasing antigen presentation to T cells, decreasing IL-12 production and inhibition of iNOS (with nitric oxide being a required factor for killing of the parasite) [16]. In addition, cell surface receptors for IgG, termed FcγRs, are required for chronic disease caused by *L. mexicana* complex parasites [9], [14]. In particular we have shown a requirement for FcγRIII [16] and IgG1 [11]. The parasite is thus able to suppress the protective Th1 IFN-γ immune response through an IgG-FcγR pathway, utilizing the host's IgG response.

**Figure 1:**
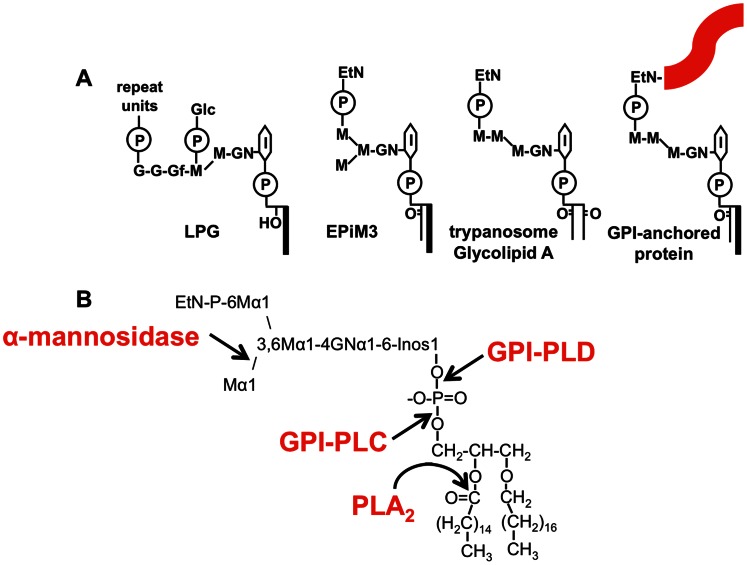
GPI structures. ***A***. Structures are shown for: *Leishmania* lipophosphoglycan (LPG); the most abundant glycoinositol phospholipid, EPiM3; the African trypanosome glycosyl phosphatidylinositol protein anchor precursor, glycolipid A; and a GPI-anchored protein such as gp63. Molecular components are abbreviated as follows: EtN, ethanolamine; G, galactose; Gf, galactofuranose; Glc, glucose; GN, glucosamine; I, myo-inositol; long line, hydrocarbon chain of acyl or alkyl group; M, mannose; and P, phosphate. Structures are modified from [17], [19]. ***B***. A more detailed structure of EPiM3 is based on [17] and shows the cleavage points for α-mannosidase, GPI-PLC, GPI-PLD, and PLA_2_. Abbreviations: Inos, myo-inositol; and other structures as in *A*. Linkages are shown. EPiM3 has predominantly C18:0 alkyl chain (*sn*-1) and C16:0 acyl chain (*sn*-2) as lipid components. EPiM3 and trypanosome glycolipid A are isomers with the difference of branched vs. linear tri-mannose chain, and alkyl-acyl vs. dimyristoyl lipids, respectively.

*Leishmania* have a wide array of glycolipids called glycosyl phosphatidylinositols (GPIs) as membrane components. Many proteins such as the promastigote surface protease, gp63, are inserted into the plasma membrane by GPI anchors rather than through trans-membrane protein domains (Fig. 1). The surface of the insect vector stage of the parasite (the promastigote) is covered with lipophosphoglycan (LPG), which consists of a GPI core with a very large phosphoglycan repeat structure (Fig. 1). Small non-protein bound GPI molecules called glycoinositol phospholipids (GIPLs) are the most abundant glycolipids on the surface of the amastigote (the mammalian host stage), and are potential antibody targets. EPiM3 is the most abundant GIPL in *L. mexicana* and likely is the molecule recognized by the mouse serum IgG, or is closely related to it in structure. EPiM3 has three mannose residues in a branched configuration [17] and is an isomer of the well-described glycolipid A from African trypanosomes, which has three linear mannose residues and a different lipid composition. Glycolipid A is the free-GPI precursor to the anchor of the variant surface glycoprotein (VSG); VSG is responsible for antigenic variation. The structures of *Leishmania* GIPLs have been determined for *L. mexicana* and several other species [17], [18], [19]. This analysis has shown species-specificity with *L. mexicana*, but not *L. major*, having ethanolamine-containing GIPLs [17], [19]; and by contrast, *L. major*, but not *L. mexicana*, having terminal galactose residues on GIPLs [20]. It has been shown that humans with cutaneous leishmaniasis have antibody responses to promastigote GIPLs [21], [22], although analysis of antibody recognition of amastigote GIPLs has not previously been performed.

It is clear that IgG in the serum of infected mice binds the surface of *L. mexicana* amastigotes and that this IgG can induce IL-10 from macrophages in vitro [16] and suppress IL-12 [23], [24]. Many groups, using sensitive metabolic labeling methods, failed to find surface proteins on *Leishmania* amastigotes that are recognized by antibodies, despite prominent IgG responses to the parasite surface [17], [25], [26]. Abundant surface proteins such as gp63, PSA2, and mPPG found on the infective insect stage of the parasite (metacyclic promastigote) are downregulated in the mammalian host stage (amastigote), as is LPG [17], [27]. As promastigotes disappear within a few days of the sandfly bite, IgG responses to these promastigote molecules are not relevant to the chronic aspects of *Leishmania* infection, which involves only the amastigote stage of the parasite. When rabbits were infected with *L. mexicana* amastigotes, they produced antibodies that bind the surface of amastigotes. These surface-binding antibodies do not recognize proteins but instead bind to GIPLs [17]. In these studies, EPiM3 (see Fig. 1), the predominant amastigote GIPL (63% of GIPLs), was strongly recognized, and was the only parasite-derived glycolipid bound by rabbit IgG. EPiM3 is quite abundant, with roughly 2×10^7^ molecules per amastigote [17]. Unfortunately, the understanding of disease and the tools needed to study the immunology of leishmaniasis are not yet available for rabbit infection.

We therefore decided to use our well-characterized mouse model to investigate whether IgG specifically recognizes the GIPLs on *L. mexicana* amastigotes, and to confirm that these particular antibodies can bind to FcγR to induce IL-10. We also began the process of identifying aspects of the structure of the immunodominant GIPL needed for antibody recognition. In addition we extended this work by determining that humans infected with *L. mexicana* also harbor anti-GIPL antibodies that can induce IL-10 from monocytes.

## Materials and Methods

### Mice

Female C57BL/6 mice (4–6 wks old) were obtained from Jackson Laboratories (Bar Harbor, ME).

### Mouse sera

Sera were obtained from tail bleeds of mice anesthetized with isofluorane at various times post-infection, or from terminal bleeds at experiment termination. Sera were stored at −20°C before use.

### Parasites and antigens

*L. mexicana* (MNYC/BZ/62/M379), *L. major* (MHOM/IL/80/Friedlin), and *L. amazonensis* (LTB004) promastigotes were grown at 27°C in Grace's medium (pH 6.3) supplemented with 20% heat-inactivated FBS, 2 mM L-glutamine, 100 U/ml penicillin, and 100 µg/ml streptomycin. For infections, stationary-phase *Leishmania* promastigotes (day 7) were washed three times in PBS and 5×10^6^ parasites (in 50 µl DMEM) were injected into the hind footpad of mice. Stationary-phase promastigote cultures of *L. mexicana* or *L. amazonensis* (day 7) were incubated at 33°C for 3 days to generate axenic amastigotes. Axenic amastigotes were passaged every 7–10 days at 1/100 into acidic Grace's medium (pH 5.5) supplemented as above. “Washed membranes” were prepared from axenic amastigotes by hypotonic lysis as described previously [16]. Briefly, axenic amastigotes were washed in PBS and then hypotonically lysed at 10^9^/ml in endotoxin-free water containing 0.1 mM N-tosyl-L-lysine-chloromethyl ketone (TLCK; Sigma-Aldrich, St. Louis, MO) and 1 µg/ml leupeptin (Sigma-Aldrich) for 5 min on ice. Then an equal volume of 0.1 mM TLCK, 1 µg/ml leupeptin, 20% glycerol was added and parasites were frozen at −80°C. Thawed lysate was washed in PBS (6100 *g*, 10 min, 4°C) to remove soluble proteins and protease inhibitors, and resuspended at 10^9^ cell equivalents/ml in PBS. This lysate was assayed for protein content by the bicinchoninic acid method (Pierce/Thermo Fisher Scientific, Rockford, IL) and brought to 1 mg/ml protein, aliquoted, and stored at −80°C. For some experiments, intracellular amastigotes devoid of mouse-derived surface antibodies were generated by infecting IC21 macrophages (ATCC, VA). IC21 cells were grown in 300 ml RPMI 1640 supplemented with 10% heat-inactivated FBS, 2 mM L-glutamine, 100 U/ml penicillin, and 100 µg/ml streptomycin, 10 mM HEPES, 1 mM sodium pyruvate in 1900 ml tissue culture flasks (T300; Becton Dickinson Labware, CA) and were infected with *L. mexicana* stationary-phase promastigotes (2×10^8^ parasites: 4.5×10^7^ cells; MOI 4.4) for 4 days at 33°C with parasite isolation by disruption of the cells ten times through a 23G needle. Parasites were then washed, counted, and frozen at −80°C. *L. mexicana* lesion amastigotes were isolated from footpads of infected C57BL/6 mice by soaking the feet in 70% ethanol, chlorhexadine, and 70% ethanol (5 min each) followed by removal of the skin and grinding the footpad with a tissue grinder in PBS with 200 U/ml penicillin, and 200 µg/ml streptomycin. Debris was removed with a low speed spin (5 min at 50 *g*) and parasites were washed three times at high speed (15 min at 1900 *g*). Amastigotes were frozen at 5×10^7^/ml in RPMI-1640 supplemented with 10% heat-inactivated FBS and 7.5% DMSO at −80°C and stored in liquid nitrogen.

### GIPL isolation

*Leishmania* GIPLs were purified as described by Winter *et al* [17]. Briefly, *Leishmania* (promastigotes or amastigotes) were washed in PBS and the pellet was extracted with chloroform∶methanol∶water (1∶2∶0.8, v/v) to precipitate proteins, and water was added to the supernatants to extract with chloroform∶methanol∶water (4∶8∶5.6). The upper methanol/water-rich phase was dried under nitrogen and re-extracted using a Folch extraction with chloroform∶methanol∶10 mM KCl (8∶4∶3); the GIPLs partition into the lower chloroform/methanol-rich phase. Samples were dried under nitrogen and resuspended in methanol for TLC analysis. Bloodstream form *Trypanosoma brucei* glycosyl phosphatidylinositols (GPIs; a kind gift of Dr. Kojo Mensa-Wilmot) were extracted using chloroform∶methanol∶water (10∶10:3), followed by extraction into the *n*-butanol phase of *n*-butanol/water (1∶1).

### IgG ELISA

Anti-GIPL ELISAs were performed by drying 2×10^7^ cell equivalents of GIPL extract onto PVC 96-well plates (Becton Dickinson Labware). Plates were blocked with 5% newborn calf serum in PBS. Samples were applied and washes were performed using PBS/0.05% tween-20. Biotinylated anti-mouse IgG1 and anti-IgG2a/c (BD Bioscience, CA) were used with detection by streptavidin-peroxidase (Jackson ImmunoResearch, PA) and ABTS substrate [2,2′-azino-bis(3-ethylbenzthiazoline-6-sulphonic acid)]. Uninfected control serum values were subtracted from infected serum values. Biotinylated 26H3T-B4 (a mouse IgG1 mAb) was detected with streptavidin-peroxidase with PBS control optical density subtracted from all results. *Leishmania*-specific IgG1 and IgG2a/c were assayed by ELISA using axenic amastigote “washed membranes” for capture, with detection as above for GIPL ELISAs. For human IgG ELISAs, biotin-goat anti-human IgG (Jackson ImmunoResearch) was used for detection.

### Monoclonal antibody preparation and biotinylation

Standard methods were used to fuse splenocytes from an *L. mexicana*-infected B6 mouse with Sp2/0 cells (ATCC) to form hybridomas. Hybridoma supernatants were screened by ELISA using axenic amastigote “washed membranes” as capture reagent, and further screened using the GIPL ELISA. Positive clones were recloned at least twice by limiting dilution in 96-well plates. Monoclonal antibodies were prepared by growing cells in DMEM supplemented with 10% heat-inactivated ultralow IgG FBS (Invitrogen, CA), 25 mM HEPES (pH 7.4), 50 µM 2-mercapto-ethanol, 2 mM L-glutamine, 100 U/ml penicillin, 100 µg/ml streptomycin, 1X non-essential amino acids (Invitrogen), 1.14 mM oxaloacetate, 0.46 mM sodium pyruvate, and 8 µg/ml rHuman insulin (Invitrogen) in bioreactor bags (Biovectra, Canada). IgG was precipitated using ammonium sulfate, dialysed against PBS, and purified with rProtein G agarose chromatography (Upstate Biotech, NY) according to the manufacturer's suggestions. IgG concentration was determined using the bicinchoninic acid method (Pierce/Thermo Fisher Scientific). 26H3T-B4 mAb was biotinylated with a 25 molar excess of sulfo-NHS-LC-biotin (Pierce/Thermo Fisher Scientific) in PBS for 4 hours at room temperature and then separated from unreacted reagent using a spin concentrator (50 kDa cutoff; Vivascience, Germany) with the equivalent of 1.2×10^6^-fold dilution with PBS.

### Thin layer chromatography and GIPL immunoblot

Thin layer chromatography (TLC) was performed by spotting samples (2×10^7^–2×10^8^ cell equivalents) on pre-baked silica gel HPTLC plates (J.T. Baker, NJ) with separation using chloroform∶methanol∶28%NH_4_OH∶1M NH_4_OAc∶water (180∶140∶9∶9∶23) in a pre-incubated glass TLC tank. Immunoblot techniques were modified from [28]. Dried plates were plasticized with 0.1% polyisobutylmethacrylate in hexane for 60 sec., then dried, blocked by spraying with 1% bovine serum albumin (BSA) in water, and then layered with 1% BSA/PBS. Washes were performed using PBS. Appropriate dilutions of serum (approx. 1/4,000 in PBS) were layered on the plate in a humidified box, and antibodies were detected using alkaline phosphatase-labeled goat anti-mouse IgG (Jackson ImmunoResearch) and then Western Blue substrate (Promega, WI). GIPL bands that reacted with mouse IgG appear purple and are detected using a flatbed scanner (HP Scanjet 4370). To visualize glycolipids, two TLC plates were run in parallel, and one plate was stained using 0.2% orcinol in water∶ethanol∶conc. H_2_SO_4_ (1∶15∶2), which makes mauve spots when baked at 115°C for 15 min. Orcinol stained plates were also scanned on a flatbed scanner.

### Enzyme digestions of GIPLs

Purified *L. mexicana* amastigote GIPLs were digested with four enzymes under the following conditions: 1) 360 U recombinant *T. brucei* GPI-PLC (a generous gift of Dr. Kojo Mensa-Wilmot) for 3 hours at 37°C in 50 mM Tris-HCl (pH 8.0), 5 mM Na_2_EDTA (pH 8), 1% Triton X-100, and 1 M potassium glutamate, with an additional 360 U added with further digestion overnight [29]; 2) human GPI-PLD (8 µl human serum) at 37°C for 2.5 hours in 50 mM Tris-HCl (pH 7.4), 10 mM NaCl, 2.6 mM CaCl_2_, and 0.1% Triton X-100, followed by 8 µl more human serum incubated for 2.5 hours, and a final 8 µl added with incubation overnight [30]; 3) jack bean α-mannosidase (added as washed ammonium sulfate suspension, 50 U/ml final; Sigma-Aldrich, MO), with samples dissolved in 100 mM sodium acetate (pH 5.0), 0.1% taurodeoxycholate and incubated overnight at 37°C followed by repeat overnight digestion with the same amount of enzyme [30]; or 100 U bee venom phospholipase A_2_ (PLA_2_, Sigma-Aldrich) in 25 mM HEPES pH 7.4, 1 mM CaCl_2_ incubated for 3 hours at 37°C, with a second digestion as before [31]. After digestion, the GIPLs were partitioned into *n*-butanol [butanol phase of butanol/water (1∶1)] before separation by TLC. To be sure that GIPLs do not run aberrantly on TLC due to residual detergent, samples were back extracted with water, and mock digestions were performed without enzyme, as a control. With PLA_2_ digestion, the aqueous phases were purified using a Sep-pak plus C18 reverse-phase syringe cartridge (Waters Corp., MA) as described previously [31]. Briefly, the cartridge was washed with 10 ml methanol followed by10 ml water, and then the aqueous GPI-containing sample (400 µl) was slowly applied. The cartridge was washed with 5 ml water, and then the GPIs were eluted with 5 ml methanol.

### Flow cytometry

Mouse IgG on the surface of amastigotes was measured using PE-rat anti-mouse IgG1 (BD Bioscience), PE-goat anti-mouse IgG (Invitrogen), and FITC-rat anti-mouse IgG2a (BD Bioscience). Staining was performed for IgG1 and IgG2a/c in different tubes to avoid the need for compensation, and to remove the possibility of fluorescence overlap. When axenic amastigotes were opsonized (4°C, 30–60 min in 50 µl volume of IgG-containing serum dilution), surface IgG was similar to that of lesion-derived amastigotes. Human IgG was detected using PE-anti-human IgG (BD Bioscience). Flow cytometry was acquired on a FACSCaliber or FACSCanto flow cytometer (BD Biosciences) and analyzed with CellQuest Pro (BD Biosciences) or FlowJo (Tree Star, Inc., OR) on an Apple Macintosh computer. For monoclonal antibody binding to axenic amastigotes, one million parasites were incubated with antibody at varying concentrations in triplicate.

### Human sera

Serum samples from Mexican patients infected with *L. mexicana* were generously provided by Dr. Ingeborg Becker of the Facultad de Medicina, Universidad Nacional Autónoma de México in Mexico City, Mexico. Four samples for each group were tested from localized cutaneous leishmaniasis (LCL), a disseminated skin disease called diffuse cutaneous leishmaniasis (DCL), and endemic controls. Anonymous random human serum that was excess material discarded from clinical samples from the Philadelphia Veterans Affairs Medical Center was used as “PA nl” and gave results similar to those of the endemic controls.

### Infection of mouse macrophages and human blood monocytes

Mouse bone marrow macrophages were prepared and infected at 10∶1 (parasites: cells) as previously described [16]. Axenic amastigotes were opsonized for 30 min. on ice with serum from infected mice, or other antibody preparations, and washed before infection of macrophages. Lipopolysaccharide (LPS) from *Escherichia coli* 0111:B4 (Sigma-Aldrich, MO) was added at 100 ng/ml. Purified anti-IL-10R (1B1.3a, a generous gift from DNAX) was added to cultures at 9 µg/ml. Human blood monocytes, provided by the University of Pennsylvania Center For AIDS Research Immunology Core, were prepared by countercurrent elutriation (>90% pure), rested overnight in 24-well plates at 2.5×10^5^/well, and stimulated with LPS and/or opsonized parasites in the same manner as mouse macrophages. IRB approval was obtained to use these de-identified human cells. For murine macrophages and human monocytes, cells were incubated for 20 hours, supernatants were frozen, and then were assayed for IL-10 after thawing. All in vitro macrophage/monocyte stimulations were performed in quadruplicate.

### IL-10 ELISA

Supernatants were assayed for IL-10 by ELISA using an antibody pair (for mouse IL-10) or a human IL-10 BDOptEIA kit (for human IL-10) according to the manufacturer's recommendations (BD Bioscience).

### Statistics

Macrophage and monocyte cultures were incubated in quadruplicate with means and SE shown for all four. The kinetics of anti-GIPL antibody production was quantitated as above, with means and SE shown for 5–13 mice per group. Human serum experiments were performed with 4 patients each of uninfected Mexican controls, LCL, and DCL patients with mean and SE shown for the group. All experiments were performed at least twice with representative data shown. Monoclonal antibody binding to axenic amastigotes was performed in triplicate with geometric mean fluorescence intensities calculated and presented as mean and SE. A student T test was used to compare groups of mice, groups of patients, or replicate samples, with *P* values shown and *P*<0.05 considered significant.

### Ethics

Animal studies were reviewed and approved by the Institutional Animal Care and Use Committee of the Philadelphia Veterans Affairs Medical Center and of the University of Pennsylvania, and were carried out in strict accordance with the “Guide for the Care and Use of Laboratory Animals” of the National Academy of Sciences, as well as all other U.S. government federal guidelines. Human serum samples and human random donor monocytes were de-identified, and therefore the protocol was deemed exempt from requirements for informed consent by the Institutional Review Boards of the Philadelphia Veterans Affairs Medical Center (FWA #00001311) and the University of Pennsylvania (FWA #00004048). The IRB of the Universidad Nacional Autónoma de México in Mexico City, Mexico also approved the serum collection and use.

## Results

### B6 mice generate an IgG response to the surface of *L. mexicana* amastigotes

**Figure 2:**
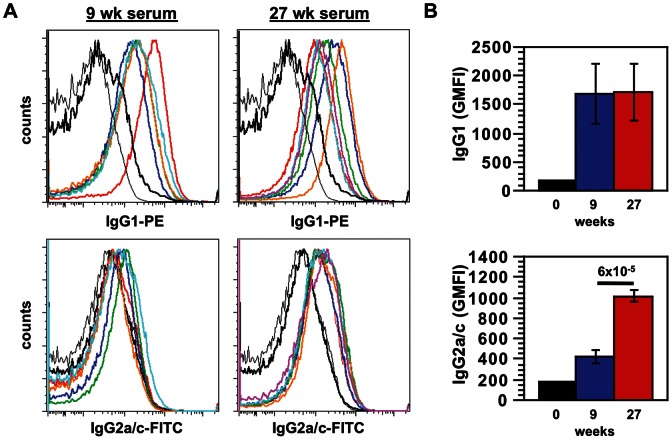
Opsonization with early and late *L. mexicana* anti-sera. Sera from mice infected with *L. mexicana* for 9 wks (n = 5) and 27 wks (n = 6) were used to opsonize *L. mexicana* axenic amastigotes. ***A***. Parasite surface mouse-IgG1 and -IgG2a/c were detected by flow cytometry. Unopsonized amastigotes (thin black), uninfected serum (thick black), and serum from infected mice (colored) are shown. ***B***. Data from *A* with Geometric Mean Fluorescence Intensity (GMFI) are shown for each time point, with mean and SE. *P* = 6×10^−5^ for the difference between 9- and 27-wk samples. Data are representative of 2 similar experiments.

We have shown by ELISA that at early times there is an IgG1 response and later an IgG2a/c response in B6 mice to parasite antigens (freeze-thawed Ag). Here we showed that this same kinetics occur with amastigote surface-binding IgG. Strong IgG1 and negligible IgG2a/c are seen at 9 wks post-infection, but both IgG1 and IgG2a/c to surface epitopes are present late in infection (27 wks) (Fig. 2A). The geometric mean fluorescence intensities (GMFI) are shown (Fig. 2B), with significant differences between 9- and 27-wk IgG2a/c binding, but no difference in IgG1 binding at the two time points. We have already shown that IgG, when bound to *L. mexicana* amastigotes, can induce IL-10 from macrophages stimulated with lipopolysaccharide [16], and that IL-10 is required for chronic disease caused by infection with this parasite [14].

### *L. mexicana*-infected mice have an IgG1 response to GIPLs

**Figure 3:**
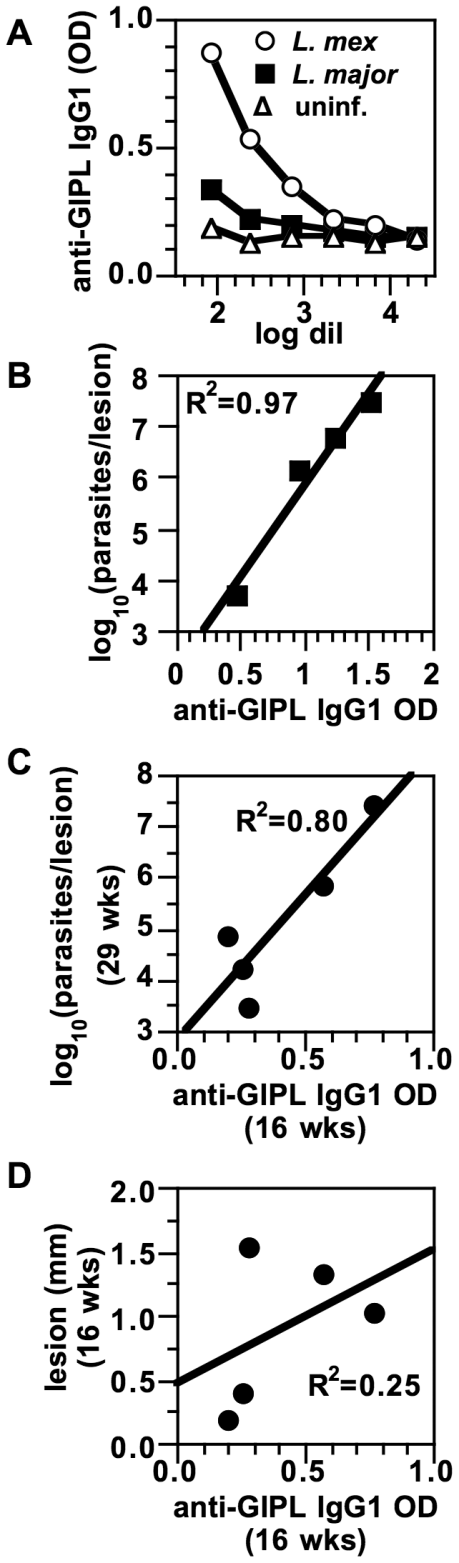
*L. mexicana*-infected B6 mice have an IgG1 response to GIPLs that is parasite species-specific and predicts parasite load. ***A***. GIPLs from *L. mexicana* amastigotes were purified and dried onto a polyvinyl chloride 96-well plate, and sera from *L. mexicana*-infected (*L. mex*), *L. major*-infected (*L. major*), and uninfected (uninf.) B6 mice were assayed for IgG1 binding by ELISA. ***B***. Serum from B6 mice infected with *L. mexicana* for 28 wks (1/15 dil) was assayed for anti-GIPL IgG1 as in *A* and correlated with log parasite loads (also at 28 wks) determined by limiting dilution. ***C***. Anti-GIPL IgG1 responses at 16 wks post-infection were correlated with parasite loads at 29 wks post-infection in the same mice. ***D***. Anti-GIPL IgG1 responses at 16 wks of infection were plotted against lesion sizes at 16 wks. Individual dots represent individual mice. The data represent two experiments with similar results. R^2^ for correlations are shown.

Using methods previously described, we extracted a class of glycolipids called glycosyl phosphatidylinositols from *L. mexicana* amastigotes [17] and bound these to a polyvinyl chloride plate for use in an ELISA. We found that serum from *L. mexicana*-infected mice had IgG1 that specifically bound *L. mexicana* GIPLs, and that there was minimal binding from the serum of *L. major*-infected mice (Fig. 3A). No anti-GIPL IgG was found in uninfected mice. A similar ELISA also detected anti-GIPL IgG2a/c in the serum of *L. mexicana*-infected mice, but at later times in infection (not shown). Note that these IgG-recognized glycolipids will be referred to as GIPLs, as they have a GPI structure (see below for more detailed structural analysis).

We found that the GIPL reactivity of serum from 28 wk-infected mice was highly positively correlated with their parasite loads (R^2^ = 0.97, Fig. 3B). This could either be due to anti-GIPL antibodies having a pathogenic effect and leading to poorer control of parasites, or conversely a consequence of higher parasite loads inducing higher IgG titers. To examine this further, we serially bled mice throughout an infection and looked for correlations between parasite loads at both 16 wks and 29 wks with various parameters such as lesion size, and IgG1 and IgG2a/c directed against GIPLs and washed membranes. Note that this infection had larger variation in parasite burdens than usual (for unknown reasons) and was used because greater variation allowed for comparisons not possible if parasite burdens were very similar in all mice. We found that the anti-GIPL IgG1 response at 16 wks correlated well with the parasite load in the same mouse at 29 wks of infection (R^2^ = 0.80, Fig. 3C). Anti-GIPL IgG1 at 16 wks did not closely correlate with lesion size at 16 wks (R^2^ = 0.25, Fig. 3D), indicating that anti-GIPL antibodies and inflammation in the lesion are not closely tied. Because of the temporal relationship, we concluded that anti-GIPL IgG1 responses earlier in infection could potentially cause increased parasite burdens later in infection due to IL-10 suppression of a Th1 response. New data indicate that *L. mexicana* parasite loads do not increase early in infection, but only increase later once the IgG1-induced IL-10 is present (Jude Uzonna, University of Manitoba, personal communication). Furthermore, there was no correlation between parasite burdens (at 29 wks) and IgG1 directed at *L. mexicana* amastigote “washed membranes” or between parasite burdens and IgG2a/c responses to GIPLs or “washed membranes” (at 16 or 29 wks, data not shown). This implies that IgG1 antibodies to GIPLs may be more important in determining parasite loads (through the FcγR-IL-10 pathway) than antibodies directed against other parasite components, such as proteins, and more important than IgG2a/c antibodies to GIPLs or other parasite molecules. This is consistent with the hypothesis that there is a strong IgG1 antibody response directed against GIPLs, but not against surface proteins, presumably because surface proteins are masked in some way. Because GIPLs are present on the amastigote surface, whereas protein targets of antibodies may reside within the parasite, antibodies to GIPLs, but not proteins, can efficiently induce IL-10 through the FcγR pathway.

**Figure 4:**
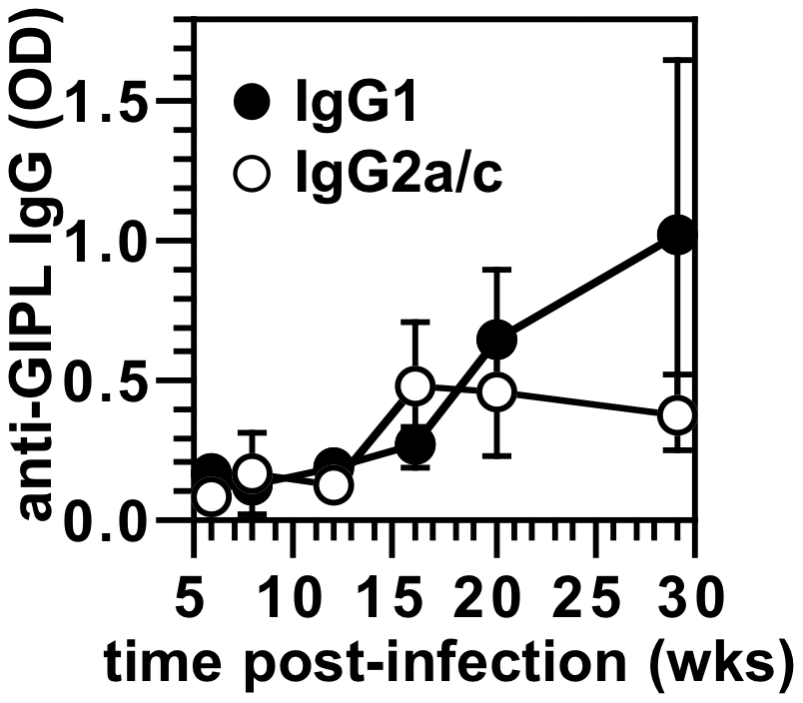
Kinetics of antibodies to GIPLs in *L. mexicana* infection. B6 mice were infected with 5×10^6^ stationary-phase *L. mexicana* promastigotes, and sera from various times of infection were assayed for anti-GIPL IgG1 and IgG2a/c by ELISA. Mean and SE for groups of mice are shown. Sera from at least 5 mice per time point are shown; data represent two experiments with similar results.

When mice were bled throughout a course of *L. mexicana* infection, we found that IgG1 recognition of GIPLs increased rapidly between 10 and 20 wks of infection (Fig. 4), consistent with the time when chronic lesions plateau in size (B6 mice) rather than resolving in strains of mice that heal (IL-10 KO and FcγRIII KO mice) [17], [27]. IgG2a/c that recognized GIPLs was also present (Fig. 4).

### IgG recognizes one GIPL spot on immunoblot

**Figure 5:**
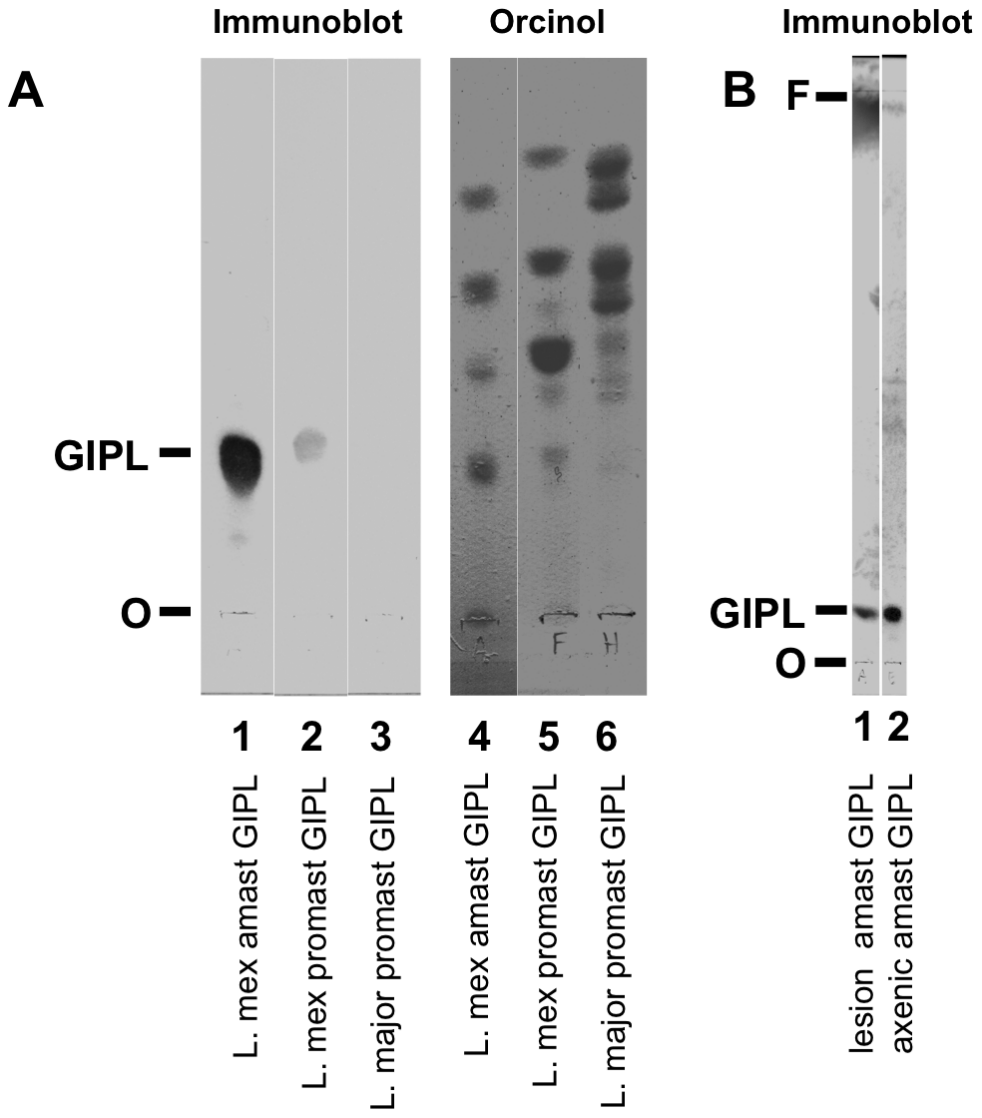
IgG recognizes a single GIPL spot on TLC immunoblot. ***A***. Purified GIPLs [2×10^8^ cell equivalents (c.e.)] from *L. mexicana* amastigotes (lanes 1 and 4), *L. mexicana* stationary-phase promastigotes (lanes 2 and 5), and *L. major* stationary-phase promastigotes (lanes 3 and 6) were separated by TLC. Lanes 1–3 were stained by immunoblot with serum from *L. mexicana*-infected mice (from 29 wks). Lanes 4–6 were stained with orcinol to visualize GIPLs. O, origin; F, front; GIPL, the GIPL recognized by anti-serum. ***B***. Immunoblot as in *A*: *L. mexicana* lesion amastigote GIPL (lane 1, 2×10^8^ c.e.), *L. mexicana* axenic amastigote GIPL (lane 2, 10^8^ c.e.). Data represent at least 12 experiments using 4 different sera, with similar results.

Next we separated the GIPLs by thin layer chromatography (TLC) and probed with anti-sera from *L. mexicana*-infected mice. We found that a single spot was recognized in *L. mexicana* axenic amastigote GIPL extract and to a much lesser extent in stationary-phase promastigotes (Fig. 5). *L. mexicana*-infected mouse serum did not recognize GIPLs from promastigotes of *L. major* (Fig. 5A), nor GIPLs from *L. major* amastigotes derived from IC-21 cells (data not shown). Uninfected mouse serum gave no binding to amastigote GIPLs in several similar experiments (data not shown). Staining with orcinol also demonstrated the strongly recognized GIPL molecule(s) in addition to other spots (Fig. 5A). These additional spots are also glycolipids, but they are not recognized by antibodies in the infected mouse sera. On immunoblot, GIPLs from lesion amastigotes had similar reactivity to GIPLs from axenic amastigotes (Fig. 5B).

### The glycolipid recognized by serum from *L. mexicana*-infected mice has a GPI structure and a branched mannose configuration

**Figure 6:**
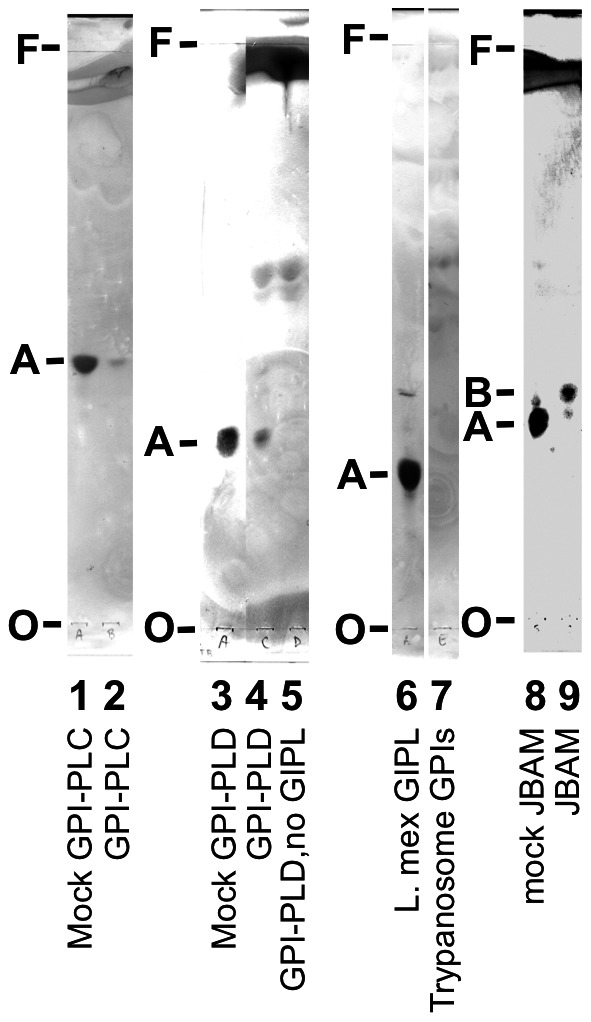
The immunodominant glycolipid recognized by *L. mexicana*-infected mouse serum is a GPI and has a branched mannose chain. *L. mexicana* amastigote GIPLs (lanes 1–4, 6, 8, 9) were digested with *T. brucei* GPI-PLC, human GPI-PLD, or jack bean α-mannosidase (JBAM), extracted with *n*-butanol, and separated by TLC. Then serum from *L. mexicana*-infected mice was used for immunoblot. In addition, *Trypanosoma brucei* GPI glycolipids were also analyzed by TLC immunoblot in comparison with *L. mexicana* amastigote GIPLs. Lane 1, 8×10^7^ c.e. GIPL, mock GPI-PLC; lane 2, 8×10^7^ c.e., GPI-PLC; lane 3, 8×10^7^ c.e., mock GPI-PLD; lane 4, 8×10^7^ c.e., GPI-PLD; lane 5, GPI-PLD without *L. mex* GIPL; lane 6, 2×10^8^ c.e. GIPL; lane 7, 2×10^8^ c.e. *T. brucei* GPIs; lane 8, 8×10^8^ c.e. GIPL, mock JBAM; lane 9, 8×10^8^ c.e., JBAM. O, origin; F, front; A, immunodominant GIPL; B, de-branched mannose product of immunodominant GIPL. Data represent at least 3 experiments with similar results.

We next digested the *L. mexicana* glycolipids with enzymes that cleave glycosyl phosphatidylinositol (GPI) structures specifically, namely trypanosome GPI-PLC and human serum GPI-PLD, and found that binding of the immunodominant glycolipid by antibodies was abolished (Fig. 6). As these enzymes, which are GPI-specific, cleave the molecule of interest (with the glycan lost into the aqueous phase which is not run on the TLC), the molecule in question must have a GPI structure, and therefore is a GIPL. See Fig. 1 for structures and enzymatic cleavage points.

We found that *Trypanosoma brucei* glycolipids, such as glycolipid A (an isomer of EPiM3, see Fig. 1), are not recognized by the anti-serum (Fig. 6). Glycolipid A, unlike EPiM3, has a linear tri-mannose structure, so we decided to determine if a branched mannose is present in the recognized *L. mexicana* GIPL and is an important part of the recognized epitope. Digestion of the GIPLs with jack-bean α-mannosidase, which cleaves terminal α-linked mannose residues, resulted in a shift to a less hydrophilic (greater relative mobility) GIPL species, which is recognized by the anti-amastigote serum (Fig. 6). Therefore, the immunodominant GIPL has a branched mannose structure like EPiM3. This experiment also shows that at least some reactivity is independent of the branched mannose structure, as both the original (labeled A) and de-branched (labeled B) structures are recognized by IgG.

### The *sn*-2 fatty acid is essential for IgG binding to the immunodominant GIPL

**Figure 7:**
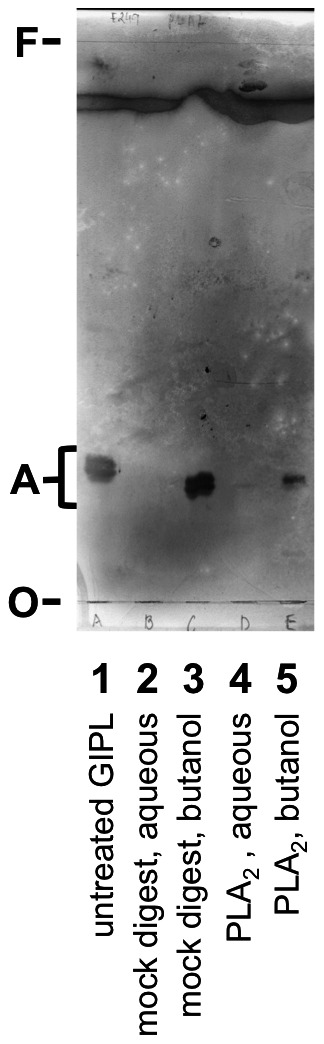
Phospholipase A_2_ abolishes IgG binding to the dominant GIPL. *L. mexicana* amastigote GIPLs (2×10^8^ cell equivalents) were digested with bee venom phospholipase A_2_ (PLA_2_) and then partitioned into water-saturated *n*-butanol and water. The aqueous phase was purified by Sep-pak C18 column as described in the Materials and Methods. Samples were separated by TLC, and an immunoblot was performed as in Fig. 5. Lane 1, untreated *L. mexicana* amastigote GIPLs; 2, aqueous phase of mock digest; 3, *n*-butanol phase of mock digest; 4, aqueous phase of PLA_2_ digest; 5, *n*-butanol phase of PLA_2_ digest. The sample in lanes 4 and 5 was not digested to completion. Data represent 4 experiments with similar results.

To help determine if the lipid portion of the GIPL is required for antibody binding, we digested *L. mexicana* amastigote GIPLs with bee venom phospholipase A_2_ (PLA_2_), which specifically cleaves the *sn*-2 fatty acid from glycolipids. Binding of the immunodominant GIPL by antibodies was greatly diminished by PLA_2_ digestion (Fig. 7), although digestion was incomplete. As lyso-glycolipids (ones with only one fatty acid or fatty alkyl group and a free hydroxyl) are significantly less hydrophobic than ones with two lipid moieties, and depending on the hydrophobicity/chain length of the lipids, can be lost in the aqueous phase during butanol-water partitioning [31], we isolated the glycolipids from the aqueous phase using a Sep-pak C18 reverse phase syringe column [31]. Despite this maneuver, there was no antibody reactivity in the aqueous phase. This indicated either that the fatty acid itself is involved in binding, or at least that the conformation of the binding site on the glycolipid depends on the *sn*-2 fatty acid.

### A mAb was developed that binds GIPLs by ELISA and the surface of parasites by flow cytometry, and induces IL-10 from macrophages

**Figure 8:**
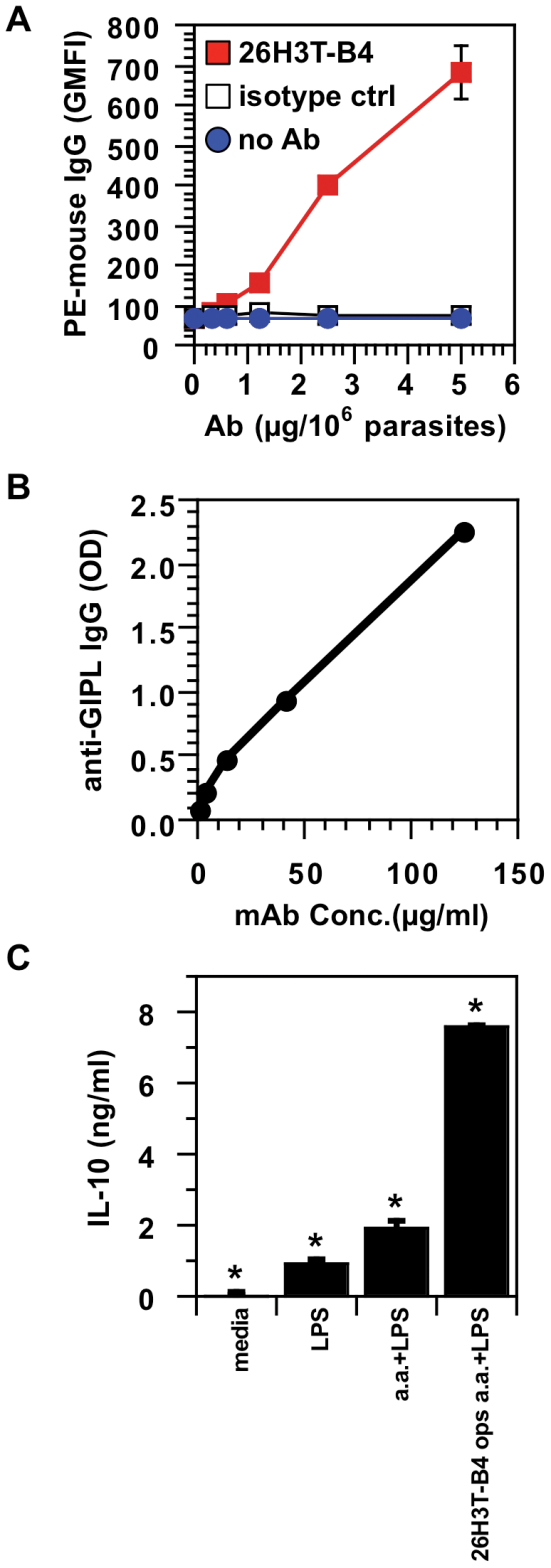
mAb binds GIPLs and the surface of amastigotes, and induces IL-10 from macrophages. ***A***. A newly developed mAb (26H3T-B4) was bound to *L. mexicana* axenic amastigotes at varying amounts per 10^6^ axenic amastigotes and detected using PE-goat anti-mouse IgG by flow cytometry, with geometric mean fluorescence intensity (GMFI) calculated for samples in triplicate; a mouse IgG1 isotype control was used at the same concentrations (isotype ctrl). Triplicate samples with no antibody (no Ab) are shown with a line for comparison. At all concentrations, GMFI were different for 26H3T-B4 vs. isotype control; GMFI for 26H3T-B4 were also different from no antibody controls. *P*<0.02 for all comparisons. ***B***. Biotinylated 26H3T-B4 was tested for GIPL binding by ELISA at varying concentrations. ***C***. Bone marrow-derived macrophages were incubated in media alone, or stimulated with lipopolysaccharide (LPS), *L. mexicana* axenic amastigotes+LPS (a.a.+LPS), or amastigotes opsonized with 26H3T-B4+LPS (26H3T-B4 ops a.a.+LPS), and then supernatants were assayed for IL-10 by ELISA. *, *P*<0.004 for all comparisons. Data represent at least 2 experiments with similar results.

In order better to assess the pathogenic nature of antibodies to GIPLs, we developed monoclonal antibodies that can bind GIPLs. They were first screened for the ability to bind *L. mexicana* “washed membranes” by ELISA and then were tested for GIPL binding. A representative IgG1 mAb (26H3T-B4) was found that efficiently binds the surface of amastigotes in a dose-response manner (Fig. 8A), indicating that the GIPL target is accessible on the parasite surface. A mouse IgG1 isotype-control mAb did not bind the surface of amastigotes, with similar values to the no-antibody control. In addition, when biotinylated, this mAb bound GIPLs very well in an ELISA (Fig. 8B), also demonstrating a dose-response relationship. We further demonstrated that the IgG1 mAb, when bound to amastigotes, induced IL-10 from murine bone marrow-derived macrophages (Fig. 8C). Unopsonized parasites and those opsonized with uninfected B6 mouse serum showed no difference in IL-10 induction [11]. Thus antibodies to GIPLs can induce an IL-10 response from macrophages.

Note that macrophages do not secrete much IL-10 when stimulated with immune complexes alone [32]. LPS is a convenient second signal, which is known not to induce much IL-10 in the absence of immune complexes. In vivo, it is likely that *Leishmania*-derived toll-like receptor ligands are important. For example the *L. mexicana* complex parasite *L. pifanoi* has a proteoglycolipid complex which, like LPS, acts through toll-like receptor 4 [33].

### Humans with *L. mexicana* infection have anti-GIPL antibodies

**Figure 9:**
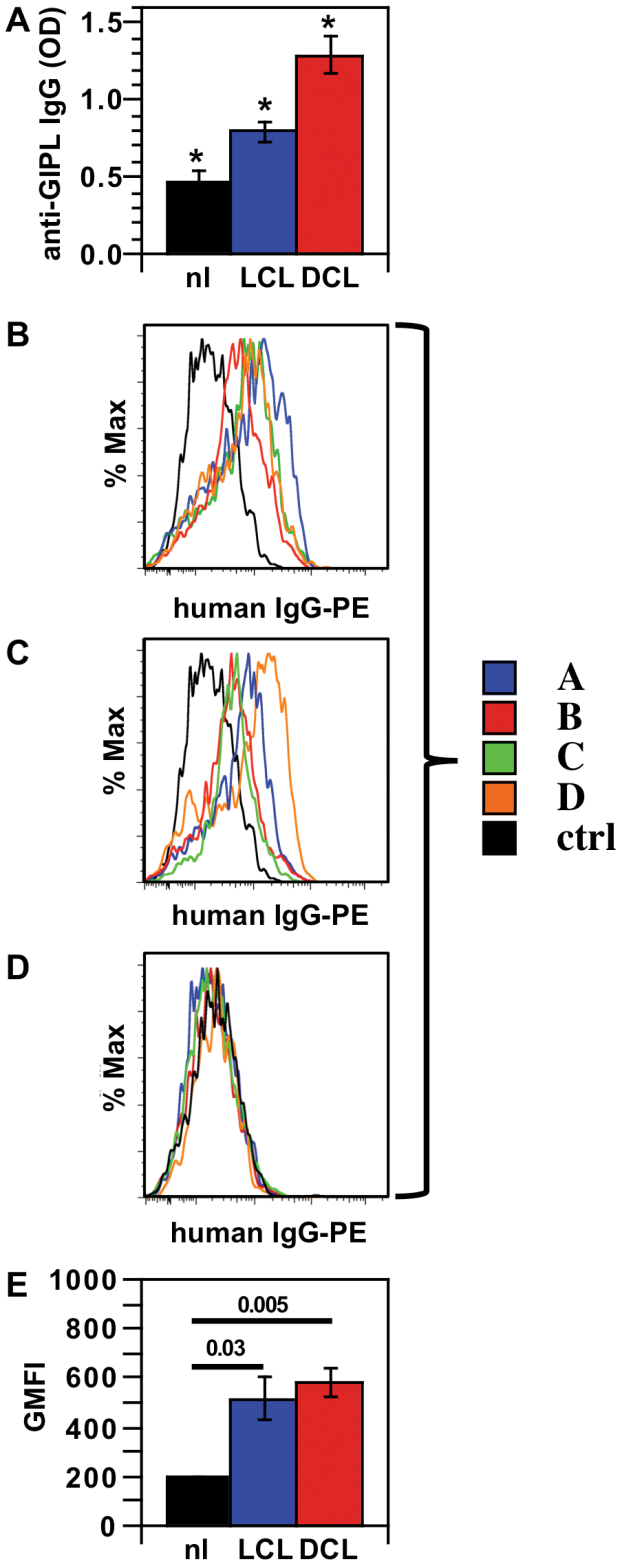
Patients with *L. mexicana* infection have anti-GIPL antibodies. ***A***. Serum from Mexican patients infected with *L. mexicana* with localized cutaneous leishmaniasis (LCL), diffuse cutaneous leishmaniasis (DCL), or Mexican controls without disease (nl) were analyzed by GIPL ELISA, and mean OD values for groups of 4 individuals are shown with SEM. *, *P*<0.01 for all pair-wise comparisons. ***B***. Sera from four DCL patients (colored lines) and a Mexican normal control (black line) were bound to *L. mexicana* axenic amastigotes, with IgG detected using PE-anti-human IgG and flow cytometry. ***C***. Same as *B*, but showing four LCL sera. ***D***. Same as *B*, but showing one Philadelphia control serum (black line) and four Mexican endemic control sera (colored lines). ***E***. A comparison of geometric mean fluorescence intensity (GMFI) of DCL, LCL, and control (nl) sera (four per group) labeled with PE anti-human IgG (data from B, C, and D analyses) is shown. Sera are designated A–D for each group of subjects. Data represent 2 experiments using the same sera with similar results.

Human infection with *L. mexicana* can cause localized cutaneous leishmaniasis (LCL), a disseminated skin disease called diffuse cutaneous leishmaniasis (DCL), and very infrequently visceral disease or mucocutaneous disease. Although high antibody titers are seen in visceral leishmaniasis, antibody titers in cutaneous leishmaniasis are not generally sensitive, with visualization of parasites from skin scrapings or biopsies being the main diagnostic tool [34]. In order to determine if human infection leads to anti-GIPL antibodies, we obtained human sera from Mexican patients with *L. mexicana* infection and LCL or DCL. We analyzed these sera using the GIPL ELISA and found that patients with LCL, and to a greater extent those with DCL, have antibodies to GIPL molecules (Fig. 9A). Background values from uninfected sera may represent low levels of natural IgG antibodies or non-specific binding. We also bound the sera from DCL and LCL patients to *L. mexicana* amastigotes and found that IgG was detectible on the parasite surface (Fig. 9B and C), with all uninfected controls (both endemic controls and one random serum sample from Pennsylvania) having lower mean fluorescence (Fig. 9D). Geometric mean fluorescence was significantly greater in DCL and LCL patients than in controls, but these groups were not significantly different from each other (Fig. 9E). Thus *L. mexicana*-infected people have antibodies that bind the parasite surface and that bind GIPLs in an ELISA format. Similar results were obtained from true *L. mexicana* amastigotes derived from the IC21 macrophage cell line as with axenic amastigote-like forms (data not shown).

### Human monocytes produce IL-10 in response to antibodies bound to *L. mexicana* amastigotes

**Figure 10:**
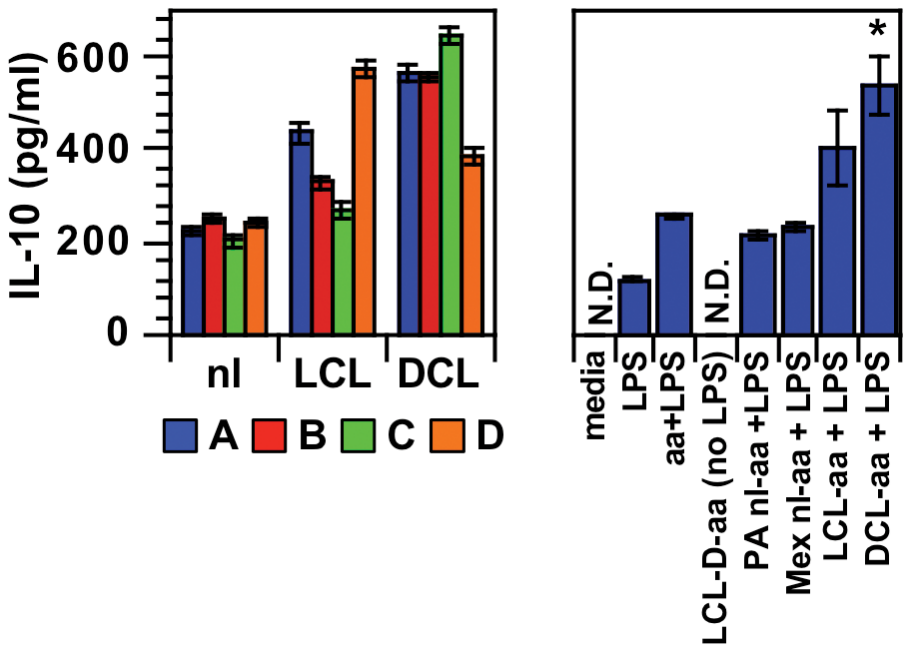
Antibodies from *L. mexicana*-infected patients induce IL-10 from human monocytes. LPS-stimulated human blood monocytes were infected with *L. mexicana* axenic amastigotes pre-opsonized with uninfected Mexican control sera (nl), or sera from patients with localized cutaneous leishmaniasis (LCL), or sera from patients with diffuse cutaneous leishmaniasis (DCL). Supernatants were harvested at 20 hours and assayed for IL-10 by ELISA. Sera from individual people are shown in different colors on the left. To allow comparisons, subjects A–D in each group have the same colors as in Fig. 9. All LCL and DCL patient sera induced significantly more IL-10 than the uninfected control mean value (*P*<0.05). Unstimulated monocytes without LPS (media) produced no detectable IL-10. LCL patient D serum-opsonized amastigotes induced no IL-10 in the absence of LPS [LCL-D-aa (no LPS)]. All parasite-infected cells stimulated with LPS made more IL-10 than LPS alone (LPS). An uninfected Pennsylvania control (PA nl-aa+LPS), the means for Mexican uninfected controls (Mex nl-aa+LPS), amastigotes opsonized with serum from LCL patients (LCL-aa+LPS), and DCL patients (DCL-aa+LPS) are also shown. Mex nl-aa+LPS, LCL-aa+LPS, and DCL-aa+LPS show the mean and SE for four patients; other samples show mean and SE for quadruplicate samples of a single condition. *, *P*<0.05 for DCL-aa+LPS vs. Mex nl-aa+LPS. N.D., none detected. Data represent 2 experiments with similar results.

Human blood monocytes were stimulated with LPS and infected with *L. mexicana* axenic amastigotes that were opsonized with human sera from leishmaniasis patients, either with DCL or LCL. IL-10 production was higher (*P*<0.05) when parasites were opsonized with all DCL sera and all LCL sera as compared with the mean value for four Mexican control sera or a Pennsylvania control serum (Fig. 10, right panel), although in one LCL patient this difference was small. Thus human IgG from both types of cutaneous leishmaniasis patients, when bound to amastigotes, can stimulate IL-10 production from human cells. No IL-10 was seen when cells were unstimulated and when infection with LCL-opsonized amastigotes was not accompanied by LPS stimulation. This extends what we have seen with mouse macrophages to the human disease state.

## Discussion

Antibodies are known to have a detrimental role in *Leishmania* infection. IgG bound to parasites can induce IL-10 from macrophages, and likely other cell types, via FcγRIII. This IL-10, in turn, suppresses the immune response to the parasite by directly decreasing iNOS expression and thereby decreasing nitric oxide, which is needed for parasite clearance. In addition IL-10 down-regulates the Th1-associated IFN-γ response, which is required to activate infected cells to make nitric oxide and kill parasites. We have recently shown that IgG1, in particular, is pathogenic in vivo in B6 mice, whereas IgG2a/c is not [11]. There have been some important gaps in our understanding of this mechanism. In particular it has been challenging to determine what the targets of these pathogenic antibodies are. Whereas much is known about surface proteins (such as gp63, PSA2, and mPPG) and a glycoconjugate, LPG, on the promastigote stage of the parasite, these are down-regulated when *L. mexicana* transforms into the intracellular amastigote stage. Because promastigotes rapidly disappear from the mammalian host, whereas antibodies do not appear for at least 4–6 wks of infection, antibodies to amastigote surface molecules are likely the most relevant in this protracted chronic infection. Several groups have failed to find surface proteins on the amastigote stage of the parasite that bind antibodies, and that could be responsible for initiating this pathway. Winter and colleagues identified a GIPL, EPiM3 (see Fig. 1 for structure), that is abundant on the *L. mexicana* amastigote surface and that is recognized by rabbit antibodies that bind the amastigote surface [17]. We therefore investigated whether GIPLs are important ligands of antibodies in our well characterized mouse model.

Using flow cytometry, TLC immunoblot, and an ELISA with unfractionated GIPLs, we have now shown that antibodies from *L. mexicana*-infected mice bind the surface of *L. mexicana* amastigotes and specifically recognize *L. mexicana* GIPLs. Presumably these antibodies can access parasites when the latter are released from the macrophage. In fact amastigotes isolated from footpad lesions are coated with antibodies, probably from the lesion fluid (data not shown). There is species specificity, as antibodies from *L. major*-infected mice do not bind these same GIPLs, and sera from *L. mexicana*-infected mice do not recognize *L. major* GIPLs. The kinetics of appearance of these anti-GIPL antibodies is consistent with the generation of chronic disease in B6 mice, as compared with healing seen in IL-10 KO and FcγRIII KO mice. We used the variation in parasite loads we occasionally see in infections to demonstrate that there is a direct correlation between anti-GIPL IgG1 responses (but not IgG2a/c responses) and parasite burdens. In fact anti-GIPL IgG1 levels measured at earlier times predict higher parasite loads later in infection, suggesting that the anti-GIPL antibodies may induce higher parasite loads through IL-10, rather than merely being caused by the higher parasite burdens. Direct evidence of pathogenicity of these antibodies is currently being studied.

We have identified a number of properties of the immunodominant GIPL molecule(s). The main recognized glycolipid has a GPI structure as it is digested by GPI-PLC and GPI-PLD, enzymes that are specific for GPI structures. In addition, like EPiM3, but unlike trypanosome glycolipid A, the immunodominant GIPL has a terminal mannose present, likely part of a branched, rather than linear, tri-mannose structure. However, the molecule generated by jack bean α-mannosidase (which removes terminal mannose residues), is still recognized by some, if not all of the antibodies in the polyclonal serum, and thus this branched mannose is not needed for IgG recognition.

In addition, recognition was abolished by PLA_2_ digestion, which removes the *sn*-2 fatty acid. Others have found that the binding of proteins to glycolipids can depend on the lipid portion of glycolipids and on the local lipid environment. *E. coli* verotoxins (VT1 and VT2c) bind to a glycosphingolipid, globotriaosylceramide b3 (Gb_3_), with different affinities based on the fatty acid length on the ceramide, with VT1 and VT2c having different optimal lengths of fatty acid chains [35]. Furthermore the length of lipid chains and degree of unsaturation, as well as the surrounding phospholipid environment (sphingomyelin/cholesterol vs. dipalmitoylphosphotidylcholine/cholesterol liposomes) can alter binding of polyclonal antibodies to glycolipids such as cerebroside sulfate [36], [37]. It is believed that the “exposure” of the glycan and its conformation may be determined by an interaction between the lipid portion of the glycolipids and the lipid bilayer or liposome environment. Thus our findings that the PLA_2_-digested GIPL (a lyso-GIPL) no longer bound antibodies (Fig. 7) extends this body of knowledge of the interaction between antibodies and glycolipids and demonstrates that the recognition process is more complex than just carbohydrate recognition.

Furthermore, we have generated mAbs to GIPLs. In particular an anti-GIPL-specific IgG1 (26H3T-B4) is able to bind isolated GIPLs and the surface of amastigotes. This mAb, when bound to amastigotes, induces IL-10 from mouse macrophages. This helps demonstrate the plausibility that anti-GIPL antibodies are important in the pathogenesis of *L. mexicana* disease in mice and will aid in future studies to directly show pathogenicity of anti-GIPL antibodies.

We have also found that humans develop antibodies that recognize the surface of amastigotes and GIPLs when infected with *L. mexicana*, whether they have DCL or LCL. Furthermore human monocytes secrete IL-10 in response to immune complexes consisting of these human antibodies and *L. mexicana* amastigotes. This shows the potential relevance of our mouse studies to human disease. This opens the door to further analysis of the human response to GIPLs, and generates several important questions: Does human IgG recognize the same GIPL structures as mice? Can we block this interaction and change the course of disease with a competing small molecule? In mice, IgG1 is pathogenic but IgG2a/c appears not to be, and may be protective [11]. By generating a vaccine that induces competing antibody isotypes that are not pathogenic, can we protect against or treat *L. mexicana* infection?
